# Simulating the Entire Clinical Process for an Implant‐Supported Fixed Prosthesis: In Vitro Study on the Vertical Implications of Implant‐Abutment Connections and Rotational Freedom

**DOI:** 10.1002/cre2.924

**Published:** 2024-07-17

**Authors:** Holger Zipprich, Stefanie Ecker, Pauline Gutmann, Kathrin Seidel, Paul Weigl, Markus Schlee, Silvia Brandt

**Affiliations:** ^1^ Department of Prosthodontics Goethe University Frankfurt Frankfurt am Main Germany; ^2^ Private Practice Schrobenhausen Germany; ^3^ Department of Postgradute Education Goethe University Frankfurt Frankfurt am Main Germany; ^4^ Private Practice Forchheim Germany

**Keywords:** assembly of implant components, implant‐abutment connection, implant‐supported fixed partial denture, in vitro experiments, positional index, rotational freedom, vertical dimension

## Abstract

**Objectives:**

The aim of this in vitro study was to investigate whether and to what extent different scenarios of rotational freedom in different IAC designs affect the vertical dimension of a three‐part fixed partial denture (FPD). At the same time, the experimental setup should simulate all clinical and laboratory steps of the implementation of such an FPD as accurately as possible.

**Material and Methods:**

Twenty identical pairs of jaw models were fabricated from aluminum, each lower‐jaw model holding two implants with conical or flat IACs. Three impressions of each model were taken to fabricate stone casts and three‐unit FPDs. Three assembly scenarios were compared for the vertical position stability they offered for these FPDs, differing by how the sequential implant components (impression posts > laboratory analogs > abutments 1 > abutments 2) were aligned with the positional index of the IAC. In this way, a total of 60 stone casts and FPDs were fabricated and statistically analyzed for changes in vertical dimension (*p* < 0.05).

**Results:**

Regardless of whether a conical/flat IAC was used (*p* > 0.05), significantly greater mean changes in vertical dimension were consistently (all comparisons *p* < 0.0001) found in a “worst‐case scenario” of component alignment alternating between the left‐ and right‐limit stop of the positional index (0.286/0.350 mm) than in a “random scenario” of 10 dentists and 10 technicians with varying levels of experience freely selecting the alignment (0.003/0.014 mm) or in a “best‐case scenario” of all components being aligned with the right‐limit stop (−0.019/0.005 mm).

**Conclusions:**

The likelihood of integrating a superstructure correctly in terms of vertical dimension appears to vary considerably more with assembly strategies than with IAC designs. Specifically, our findings warrant a recommendation that all implant components should be aligned with the right‐limit stop of the positioning index.

## Summary

**What is Known:**

- Tiny vertical deviations can result in substantial clinical problems.
- Little is known about the impact of implant‐abutment connection (IAC) designs and rotational freedom on the vertical dimension of implant‐supported restorations.
- Past experimental setups, rather than simulating the entire clinical process from impression‐taking to intraoral delivery, have remained confined to specific steps.

**What This Study Adds:**

- A comprehensive experimental setup is introduced to analyze the vertical impact of IAC designs and rotational freedom on implant‐supported fixed restorations.
- We can recommend a consistent strategy of assembling implant components that will increase the chances of maintaining a correct vertical dimension.

## 1 Introduction

Dental implants are a successful and time‐tested treatment option, with a spectrum of indications ranging from single‐tooth replacement to full‐mouth rehabilitation (Buser, Sennerby, and De Bruyn 2017; Jung et al. 2012). Despite high survival rates, they continue to be associated with biological, mechanical, and esthetic complications (Jung et al. 2012). The implant‐abutment connection (IAC), in particular, is a major and error‐sensitive source of biological complications, (Lemos et al. 2018; Saidin et al. 2012) mechanical complications, (Theoharidou et al. 2008), and imprecise vertical dimensions of superstructures (Barbosa et al. 2010; Semper, Kraft, et al. 2010). Based on the orientation of the implant‐abutment interface, a fundamental distinction is generally made between conical or flat (=butt‐joint) internal IACs. Which of these two connection geometries is superior continues to be up for debate (Lemos et al. 2018; Shadid et al. 2013).

Conical IACs are characterized by a three‐dimensional taper of the implant‐abutment interface. They have been developed to achieve a frictional fit that offers high sealing capacity (Semper, Heberer, et al. 2010; Zipprich et al. 2016, 2018) and superior mechanical stability (Hansson 2003). These conical connections carry a low risk of screw loosening (Levine et al. 1999). While they are today's IAC of choice, (Yi et al. 2023) some authors have questioned the ability of implant‐level impressions taken from conical‐IAC implants to reliably yield superstructures with precise vertical dimensions (Semper, Heberer, et al. 2010; Semper‐Hogg et al. 2013). By comparison, flat IACs are disadvantageous by involving more micromovement, less sealing capacity, and no self‐inhibition (due to their clearance fit; see next paragraph) while offering the advantage of less rotational freedom (Zipprich et al. 2016, 2018; Romanos et al. 2016).

In a conical IAC, the horizontal and vertical end positions are predefined by the specific geometry of the interface, whereas the vertical end position is presumably less well defined, and hence inaccurate, involving a risk that the abutment may sink into the cone. In a flat IAC, the vertical end position is predefined by the horizontal implant shoulder and the horizontal end position by the parallel walls of the patrix (abutment) and matrix (implant), which invariably feature a clearance to prevent jamming. All flat IACs, in addition, feature a positioning index to define the rotational position of the abutment relative to the implant. Conical IACs may or may not feature such an index.

The rotational stability offered by these positional indices will vary with their specific geometric designs and manufacturing tolerances (Semper et al. 2009). It has been suggested that such “built‐in” rotational freedom between implants and abutments, by affecting the accuracy of implant‐level impressions, might give rise to incorrect positioning of superstructures (Binon 1995; de Barros Carrilho, Dias, and Elias 2005). Alternatively, one might hypothesize that errors in transferring abutment positions may result not so much from inherent rotational freedom as from rotational variations introduced in the process of assembling the components.

Position stability in the horizontal plane is essential, as the implant components get dis‐ and reassembled several times during the prosthetic workflow, which carries a risk of accumulating errors (Gallucci, Bernard, and Belser 2005; Nelson, Hildebrand, and Mehrhof 2008). The existence of rotational freedom between a right and left stop in flat IACs arises from the aforementioned need to fabricate the positional index with a clearance, and considering that the error can also increase with the number of abutments, it is clear that inaccuracies of the aforementioned type may be more pronounced with larger‐span restorations than with single‐tooth restorations.

Several reports are available on rotational freedom and on how the act of assembling the components may affect rotational positioning (Semper, Heberer, et al. 2010; Semper‐Hogg et al. 2013; Romanos et al. 2016; Semper et al. 2009; Binon 1995; Binon and McHugh 1996). However, all of these efforts have focused on specific steps rather than introducing a comprehensive experimental setup accounting for all potential modifiers by simulating the entire clinical process from impression‐taking to intraoral delivery of the implant‐supported restoration. As a consequence, the precise impact of rotational freedom on the vertical dimension of definitive restorations is currently unknown.

We, therefore, designed an in vitro study into whether, and to what extent, different scenarios of rotational freedom in different IAC designs would affect the vertical dimension of a three‐unit fixed partial denture (FPD), simulating in a comprehensive experimental setup all clinical and laboratory steps of implementing such an FPD as closely as possible. Both conical and flat IACs were to be analyzed for position stability and vertical‐dimension changes. As null hypotheses, it was assumed that no difference in vertical‐dimension changes would be observable between replicas of the same FPD featuring (1) abutments at two different index positions and (2) different IAC designs (conical vs. flat).

## 2 Materials and Methods

**Figure 1:**
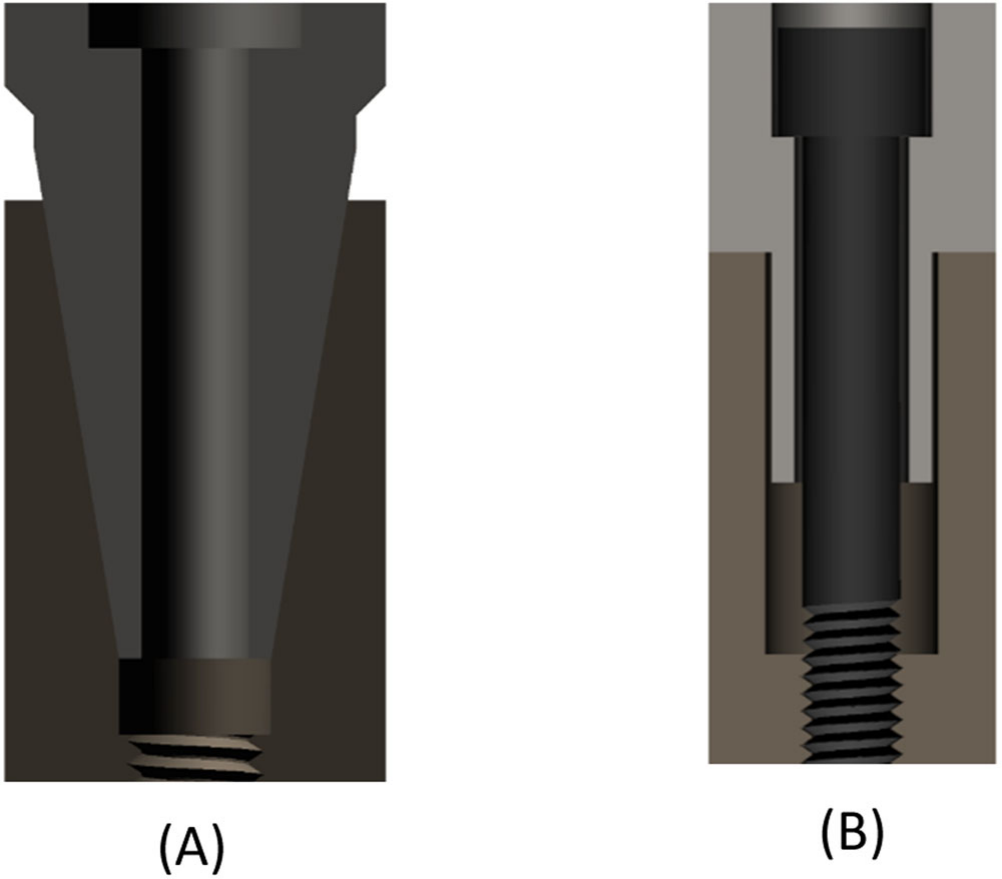
Implant‐abutment connection (IAC) designs: (A) conical and (B) flat (butt joint).

**Figure 2:**
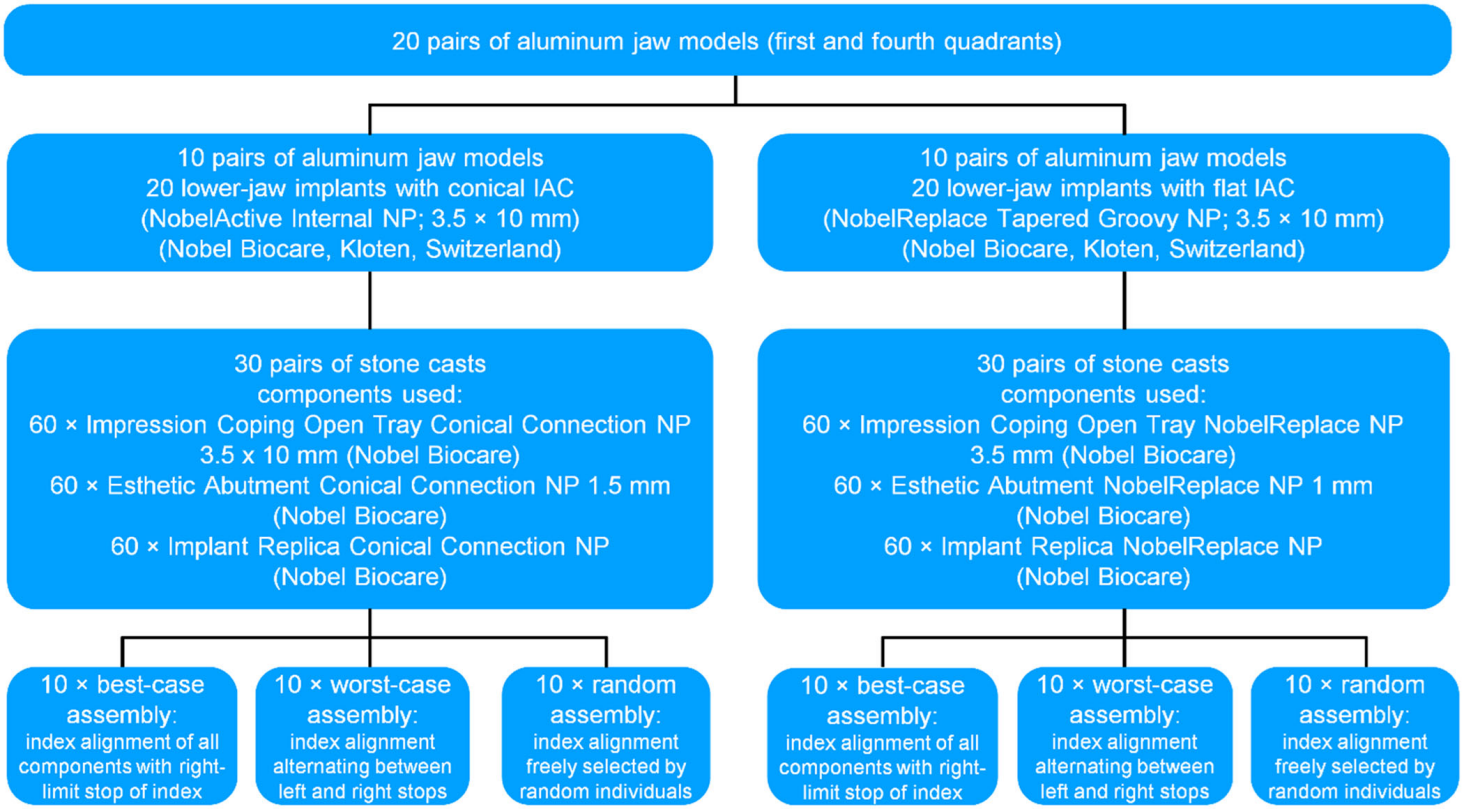
Process chart of the experimental setup. IAC, implant‐abutment connection.

Both of these IAC designs (conical and flat) are illustrated in Figure 1, and a process chart of the experimental setup used for this study is shown in Figure 2.

There are no human subjects, human data, tissue, or animals in this study; therefore, ethical approval is not applicable to this article.

### 2.1 Aluminum Models and Embedded Implants

**Figure 3:**
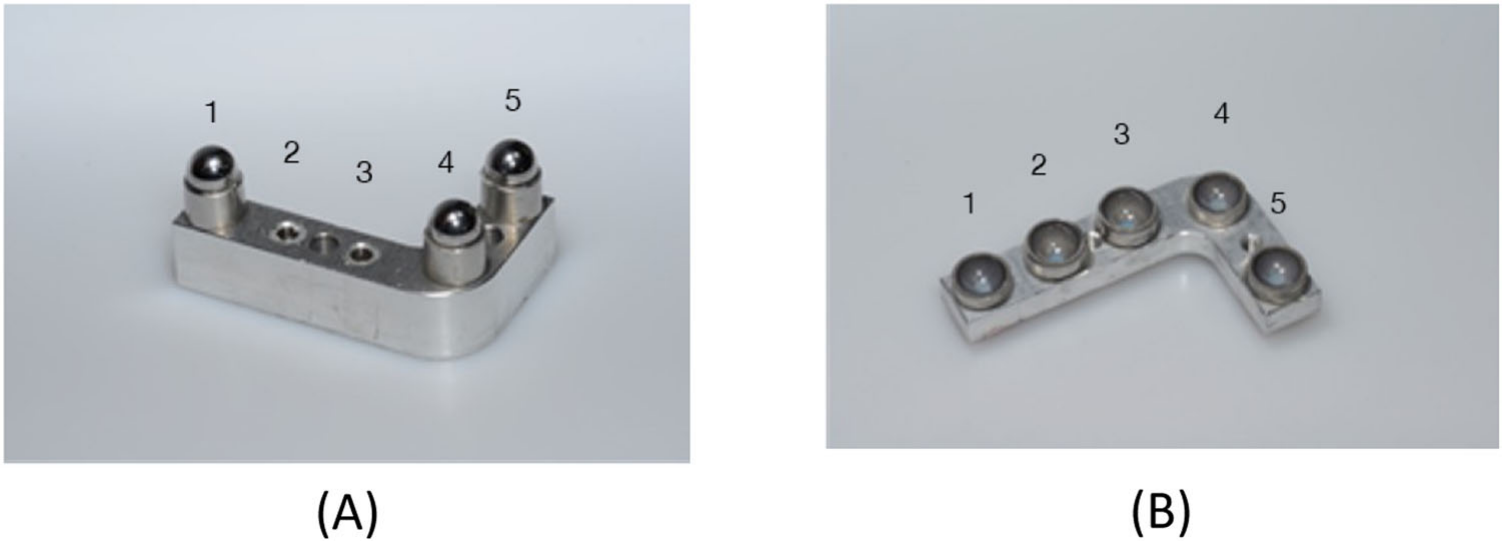
(A) Aluminum model of the lower‐jaw quadrant, fitted with stainless‐steel balls (spheres) in positions 1, 4, 5 and with implants in positions 2, 3. (B) Aluminum model of the upper‐jaw quadrant, fitted with five stainless‐steel concavities (hemispheres) to accommodate the lower‐jaw structures.

To simulate the clinical process as closely as possible, all specimens were fabricated in pairs representing the first and fourth jaw quadrants in the upper and lower jaw, respectively. Twenty of these pairs were fabricated from aluminum, and two implants were embedded at positions 2 and 3 of each lower‐jaw specimen (Figure 3A). In addition, three simulated lower‐jaw teeth were glued to positions 1, 4, and 5. In other words, a total of 40 implants were incorporated in the 20 lower‐jaw models. Implants with a 12° conical IAC (NobelActive Internal NP, 3.5 mm in diameter and 10 mm in length; Nobel Biocare, Kloten, Switzerland) were used in 10 and implants with a flat IAC (NobelReplace Tapered Groovy NP, 3.5 mm in diameter and 10 mm in length; Nobel Biocare) in the other 10 of these models. Three stainless‐steel balls (spheres) were included to simulate teeth in positions 1, 4, and 5 adjacent to both implants (Figure 3B).

### 2.2 Simulation of the Intermaxillary Relationship

Occlusion between the aluminum model pairs was simulated as follows. For positions 1, 4, and 5 of the lower‐jaw model (see Figure 3A), hemispheric concavities of stainless steel were fabricated and sandblasted to serve as housings for sandblasted stainless‐steel balls (precision spheres). These three spheres were definitively glued into the housings with a two‐component epoxy resin adhesive (Uhu Plus Endfest; Bolton Adhesives, Rotterdam, Netherlands) to serve as simulated lower‐jaw teeth adjacent to the implants incorporated in positions 2 and 3. Accurate counterparts were again established in the form of stainless‐steel housings (hemispheric concavities). After gluing these concave surfaces to the pristine half of the precision spheres (i.e., the simulated lower‐jaw teeth) using a composite resin for temporary restorations (Protemp; 3M, Maplewood, MN, USA), the bond was broken with the resin still in a fluid state. The three concave housings could now, with the resin residue in the concavities being an accurate impression of the simulated lower‐jaw teeth, be used as simulated upper‐jaw teeth (see Figure 3B). As these were being fixated to the upper‐jaw aluminum model, the lower‐jaw model was temporarily fitted with simulated teeth also in position 2 and 3, so that an accurate relationship of the upper‐jaw model could be established in occlusion with the lower‐jaw model.

### 2.3 Impression‐Taking for Casts and Superstructures

**Figure 4:**
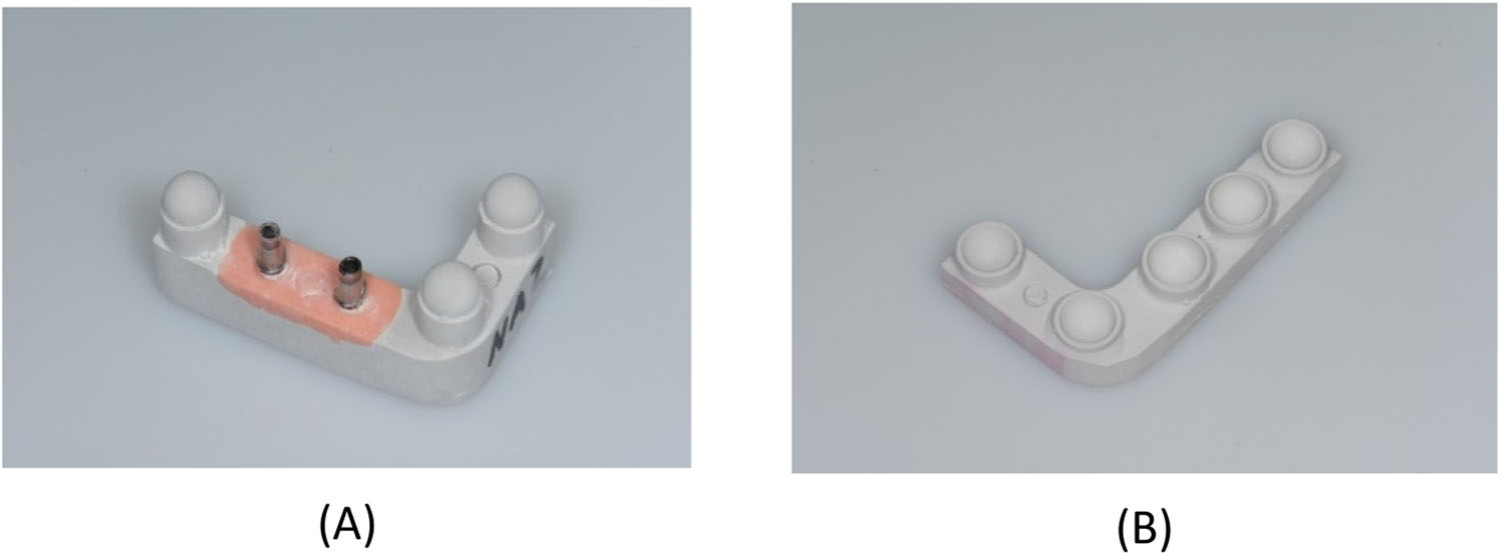
Stone casts obtained from an aluminum pair: (A) lower jaw with abutments and (B) upper jaw.

The process was continued by attaching an impression post to each of the implants in the 20 lower‐jaw models, using a suitable product for conical (Open Tray Conical Connection P 3.5 × 10 mm; Nobel Biocare) or flat (NobelReplace NP 3.5 mm; Nobel Biocare) configurations. In this way, an open‐tray impression of each model was taken in polyether (Impregum Penta; 3M, Neuss, Germany). Simulating a clinical sequence of events, the next step was to fabricate casts of type‐IV dental stone (super‐hard plaster) by hand‐tightening laboratory analogs (Conical Connection NP or NobelReplace NP; Nobel Biocare) matched to the impression posts in the conical or flat IAC configurations. Figure 4 shows one resultant pair of stone casts, with abutments already attached to the laboratory analogs.

### 2.4 Experimental Scenarios of Component Assembly

**Figure 5:**
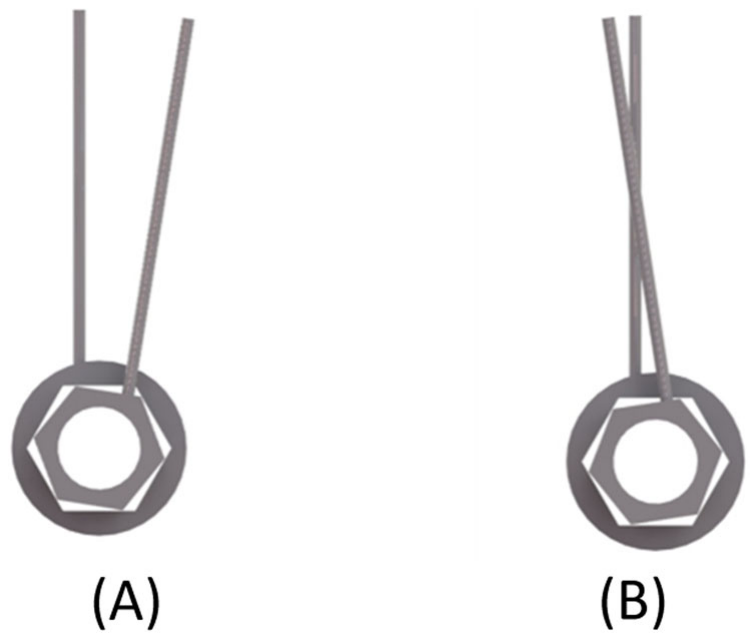
(A) Implant component aligned with the right‐limit stop of the positional index. (B) Implant component aligned with the left‐limit stop of the positional index.

While the basic impression technique that has just been outlined was used throughout the study, the exact details of arriving at the eventual stone casts did differ in that three experimental groups were formed to simulate different scenarios of attaching impression posts, laboratory analogs, and abutments:

- *Best‐case assembly scenario*. In this scenario, all components (impression posts, laboratory analogs, and abutments) were assembled in alignment with the right‐limit stop of the positional index (Figure 5A).
- *Worst‐case assembly scenario*. In this scenario, the impression posts were aligned with the left‐limit stop of the index (Figure 5B). The laboratory analog was then aligned with the right‐limit stop, and the abutment once again with the left‐limit stop. Upon fabrication of the FPD, the abutments were removed from the stone cast and replaced in alignment with the right‐limit stop.
- *Random assembly scenario*. Ten dentists with varying levels of theoretical and practical experience in implant dentistry were selected to assemble the impression posts and the abutments on the aluminum models. Ten technicians with varying experience attached the laboratory analogs and abutments on the stone casts.

### 2.5 Fabricating Casts and Connecting Abutments

A total of 60 stone casts were fabricated in this way. Once completed, abutments were hand‐tightened to the laboratory analogs, again using different products on the analogs with conical (Esthetic Abutment Conical Connection NP 1.5 mm; Nobel Biocare) versus on those with flat (Esthetic Abutment NobelReplace NP 1 mm; Nobel Biocare) IACs. The rationale for using “esthetic” abutments was to simulate customized (e.g., angulated or CAD/CAM) scenarios, as rotational freedom would not be an issue with prefabricated abutments offering an occlusal behavior of strict rotational symmetry. Also, since abutment preparation will affect the vertical‐dimension changes investigated in this study, it was important to use abutments of the same manufacturer that feature an identical occlusal design and can reasonably be expected to involve the same manufacturing tolerances.

### 2.6 Fabricating Three‐Unit Implant‐Supported FPDs

**Figure 6:**
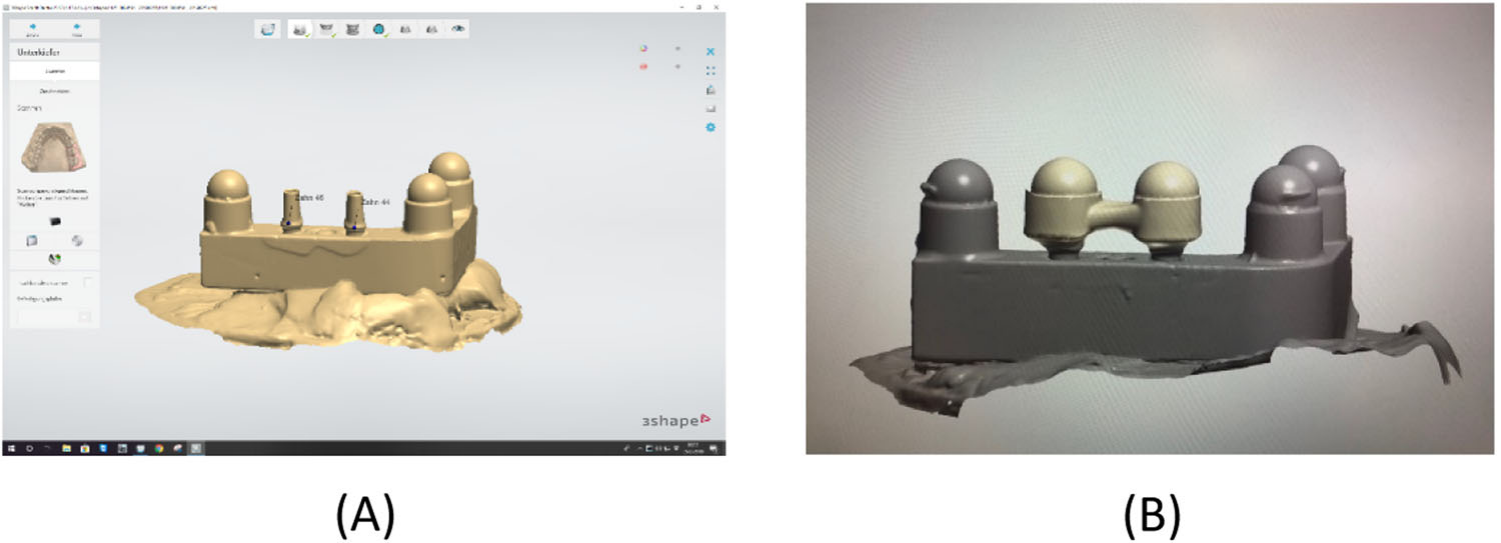
(A) Scan of a lower‐jaw stone cast with abutments (E3 Dental Lab; 3Shape). (B) Bridgework designed in CAD software (Dental System; 3Shape).

**Figure 7:**
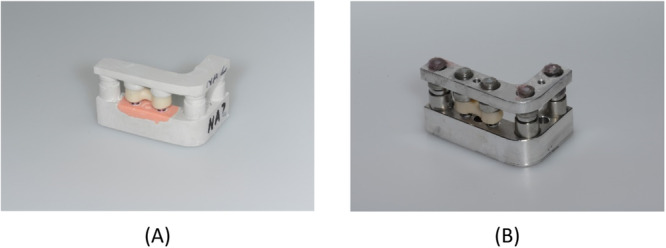
(A) Pair of stone casts with lower‐jaw FPD in situ and occlusion established. (B) Pair of aluminum models with lower‐jaw FPD in situ and occlusion established.

The next step was to fabricate 60 three‐unit implant‐supported FPDs for these stone casts. First of all, each lower‐jaw cast and abutment was coated with a non‐reflective powder (Cerec Optispray; Dentsply Sirona, Bensheim, Germany). Then the surface topography was captured by a scanner (E3 Dental Lab; 3Shape, Copenhagen, Denmark) as shown in Figure 6A, the bridgework designed in CAD software (Dental System; 3Shape) as shown in Figure 6B, and the resultant STL data transferred to a CAM unit (Ceramill Motion 2; Amann Girrbach, Pforzheim, Germany) for subtractive milling the FPD from a monolithic zirconia blank (Ceramill Zolid PS 2; Amann Girrbach). The superstructure was designed with convexities (spheres) on both abutments to engage the stainless‐steel concavities (hemispheres) in the opposing upper‐jaw cast. After 12 h of sintering (Ceramill Therm 3; Amann Girrbach), the intaglio surfaces of the restoration were sandblasted with corundum (110 µm, 2.5 bar, 10 cm) and steam‐cleaned. Subsequently, the FPDs were first placed on the stone models simulating laboratory procedures (Figure 7A) and, at a later stage, transferred to the underlying aluminum model, thus simulating clinical delivery (Figure 7B). Measurements taken during both of these stages are described in detail below.

### 2.7 Measuring Differences in Vertical Dimension

A “virtual occlusal plane” was obtained by scanning the lower‐jaw spheres and the matching upper‐jaw housings in positions 1, 4, and 5 using a 3D coordinate measuring machine (Derby 454 3D; Hexagon Aicon Etalon, Braunschweig, Germany). This machine, according to the manufacturer, has a tolerance of ±0.0035 mm. Also, the room temperature was constant enough (22°C ± 1°C) to rule out an impact of temperature variations on the measurements.

**Figure 8:**
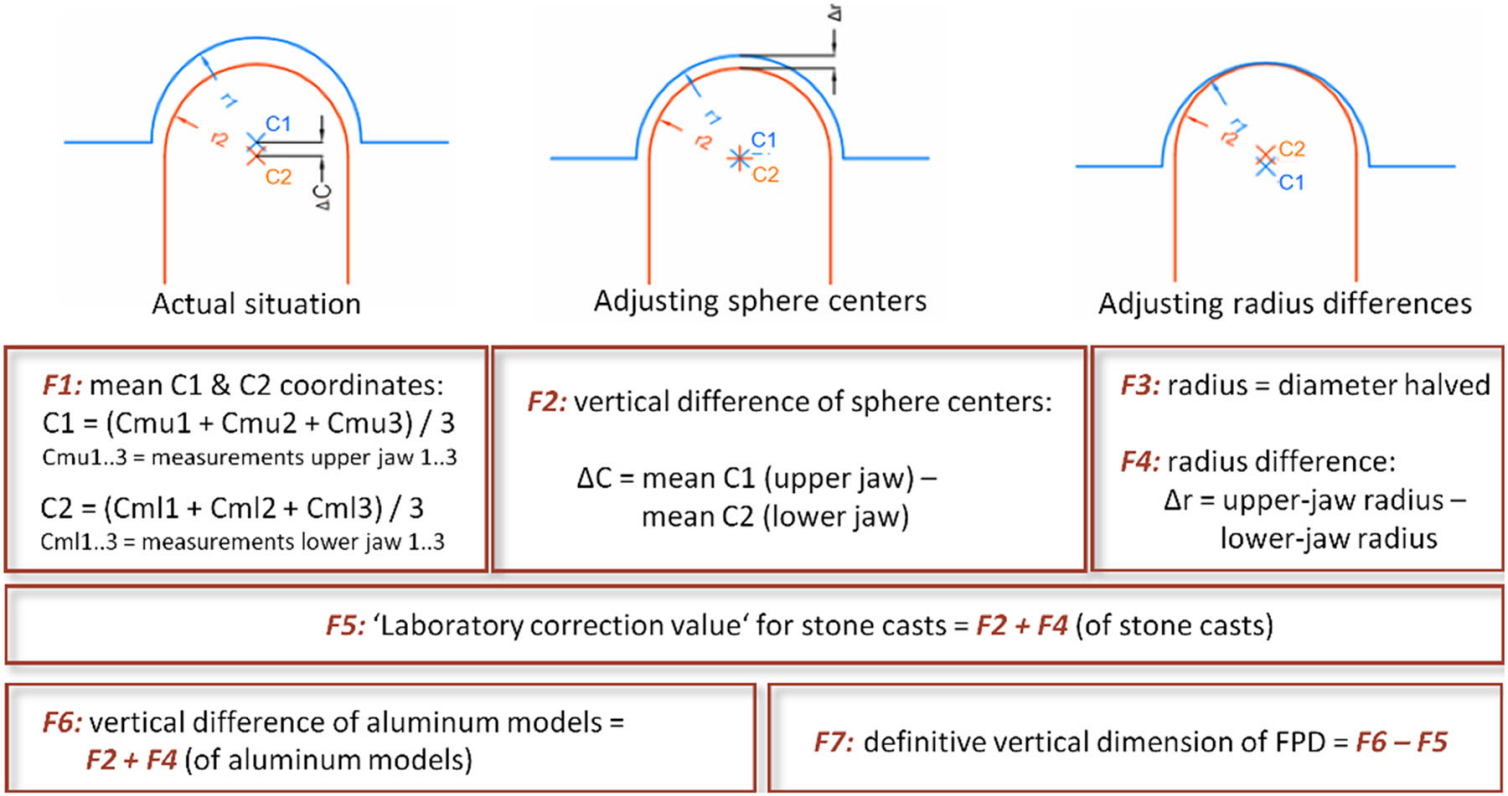
Operations to calculate both the “laboratory correction value” for the stone casts and the final vertical position of the FPD on the aluminum model. Both the convex lower‐jaw structures (i.e., the simulated FPD abutments and their adjacent teeth) and the concave upper‐jaw structures (i.e., the hemispheric housings) can be pictured as “spheres” for these geometrical calculations. C1: center of upper‐jaw sphere; C2: center of lower‐jaw sphere; r1: radius of upper‐jaw sphere; r2: radius of lower‐jaw sphere.

Figure 8 illustrates how these scans and the ensuing measurements were performed. Note that both the convex lower‐jaw structures (i.e., the simulated FPD abutments and the adjacent teeth) and the concave upper‐jaw structures (i.e., the hemispheric housings) can be pictured as “spheres” for these geometrical calculations. The scans comprised 10 surface points of each of these spheres, were performed in triplicate, and the three results were then averaged (see Figure 8, F1). On this basis, the center and diameter/radius of each sphere were calculated. Next, two jaw‐specific reference planes were obtained by connecting the three‐sphere centers in positions 1, 4, and 5, and superimposing these jaw‐specific planes yielded the required “virtual occlusal plane.”

**Figure 9:**
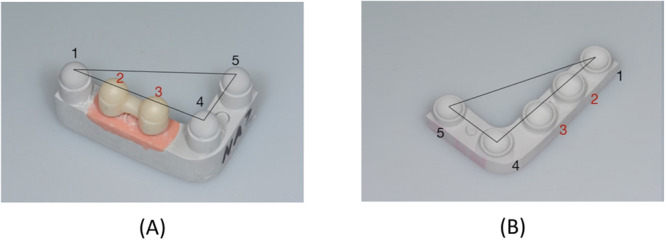
Establishing a reference plane on the stone casts (positions 1, 4, 5) and measuring the Z positions of the FPD based on positions 2 and 3: (A) in the lower jaw and (B) in the upper jaw.

**Figure 10:**
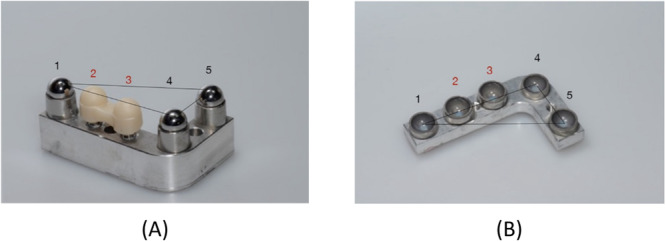
Establishing a reference plane on the aluminum models (positions 1, 4, 5) and measuring the Z positions of the FPD based on positions 2 and 3: (A) in the lower jaw and (B) in the upper jaw.

Both the stone casts and their underlying aluminum models were subjected to this procedure (Figures 9 and 10), followed by the actual measurement (again performed on both the casts and their underlying models) of determining along the *Z*‐axis the distance between the upper‐ and lower‐jaw sphere centers in positions 2 and 3 (i.e., the abutment positions of the FPD) from this “virtual occlusal plane.” To this end, abutments were connected to the aluminum models (at 35 Ncm using a torque ratchet; Nobel Biocare) and the respective FPDs temporarily cemented onto them at 50 N (TempBond; KerrHawe SA, Bioggio, Switzerland).

Note that the real‐life clinical process for an FPD would involve subtractive and/or additive adjustments (using abrasive instruments and/or ceramic firing, respectively) in the dental laboratory with the casts mounted in an articulator. However, any such steps would have modified the shape of the precision spheres, making it impossible to reliably capture their center. Instead, occlusal adjustments of this type were simulated by obtaining a “laboratory correction value” from the measurements based on the distance of the sphere centers and of the radii in positions 2 and 3 (i.e., the abutment positions of the FPD) from the “virtual occlusal plane” (see Figure 8, F5). Hence the true difference in vertical dimension was calculated from the difference between the vertical dimension on the aluminum model and the “laboratory correction value.”

### 2.8 Statistical Analysis

Mean values and standard deviations (SD) were calculated from the data obtained in all groups. Comparisons were performed by unpaired *t*‐testing, Mann–Whitney *U*‐testing, or paired *t*‐testing with appropriate statistical software, including R 3.6.1, multicomp 1.4‐10, nlme 3.1‐140, and plotrix 3.6‐3 (available at https://cran.r-project.org). Differences were considered statistically significant at *p* < 0.05.

## 3 Results

Two factors were analyzed in this study for how they would impact the vertical‐dimension changes of implant‐supported three‐unit FPDs: IAC design (conical or flat) and rotational stability. Table 1 gives a detailed overview of the mean as well as minimum and maximum values of vertical‐dimension change measured in positions 2 and 3. This is where the two implants were located in the lower‐jaw models.

**Table 1:** Vertical‐dimension changes in positions 2 and 3.

Assembly scenario	Connection type	Position 2 (mm)			Position 3 (mm)		
		Mean	Min	Max	Mean	Min	Max
Best case	Conical	−0.023	0.023	−0.073	−0.016	0.030	−0.073
Best case	Flat	0.013	0.102	−0.071	−0.003	0.085	−0.106
Worst case	Conical	0.197	0.444	−0.005	0.376	0.563	0.163
Worst case	Flat	0.293	0.710	−0.009	0.407	0.924	0.054
Random	Conical	−0.002	0.159	−0.098	0.007	0.332	−0.113
Random	Flat	0.013	0.132	−0.077	0.016	0.091	−0.055

**Figure 11:**
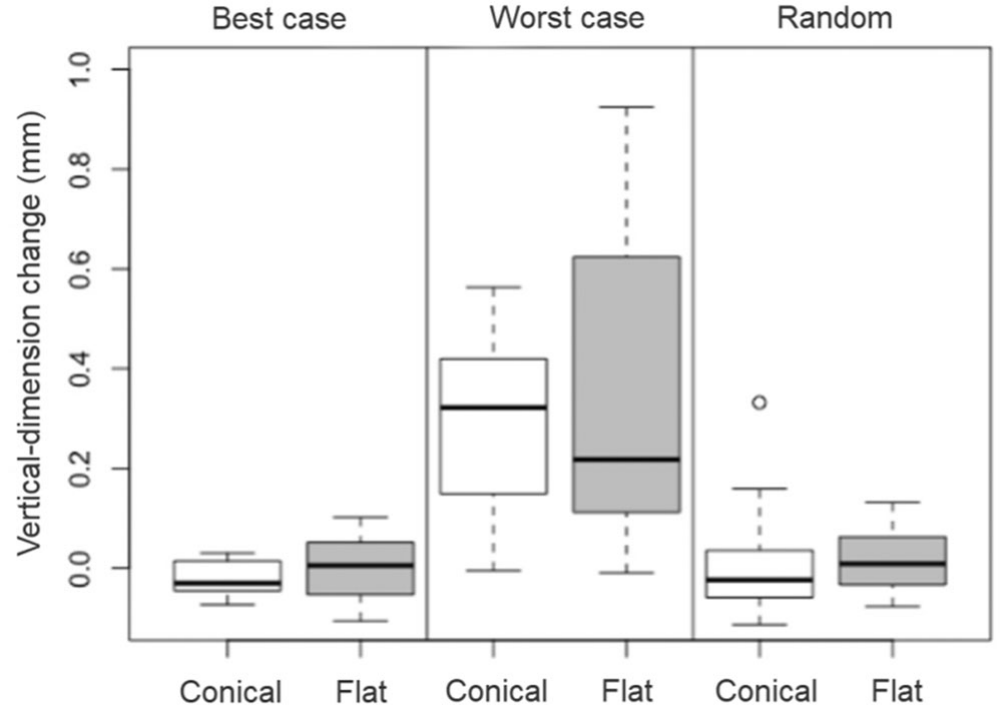
Box plot of vertical‐dimension changes in the three assembly groups.

The mean values and standard deviations for the overall changes in vertical dimension are given in Table 2 and illustrated in Figure 11. Greater changes were noted in the worst‐case assembly experiments both for the conical (0.286 mm) and the flat (0.350 mm) IACs than in the best‐case experiments (−0.019 or 0.005 mm, respectively) and the random experiments (0.003 or 0.014 mm, respectively).

**Table 2:** Vertical‐dimension changes overall.

Assembly scenario	Connection type	Vertical displacement (mm)
Best case	Conical	−0.019 ± 0.034
Best case	Flat	0.005 ± 0.061
Worst case	Conical	0.286 ± 0.180
Worst case	Flat	0.350 ± 0.300
Random	Conical	0.003 ± 0.102
Random	Flat	0.014 ± 0.062

*Note:* Data expressed as mean values ± SD.

As apparent from Table 3, significant differences between the assembly scenarios were noted for conical IACs in the worst‐case versus the random and versus the best‐case group (2× *p* < 0.0001), as well as for flat IACs in the worst‐case versus the random and versus the best‐case group (2× *p* < 0.0001). Significant differences were not observed for conical IACs in the best‐case versus the random group (*p* = 0.998) and for flat IACs in the best‐case versus the random group (*p* = 1.000).

**Table 3:** Intergroup differences based on connection types.

Connection type	Comparison between assembly scenarios	*p* value
Conical	Best case vs. random	0.998
Conical	Worst case vs. random	0.0001*
Conical	Worst case vs. best case	0.0001*
Flat	Best case vs. random	1.000
Flat	Worst case vs. random	0.0001*
Flat	Worst case vs. best case	0.0001*

* Differences that are statistically significant.

The comparisons based on IAC designs in Table 4 reveal no significant differences in vertical‐dimension changes for conical versus flat IACs, whether in the best‐case (*p* = 0.996), the worst‐case (*p* = 0.776), or in the random (*p* = 1.000) experiments.

**Table 4:** Intergroup differences based on assembly scenarios.

Assembly scenario	Comparison between connection types	*p* value
Best case	Conical vs. flat	0.996
Worst case	Conical vs. flat	0.776
Random	Conical vs. flat	1.000

Other comparisons across connection types and assembly scenarios are listed in Table 5. Significant differences emerged for conical IACs in the worst‐case versus flat IACs in the best‐case experiments (*p* < 0.0001), for conical IACs in the random versus flat IACs in the worst‐case experiments (*p* < 0.0001), for conical IACs in the best‐case versus flat IACs in the worst‐case experiments (*p* < 0.0001), and for conical IACs in the worst‐case versus flat IACs in the random experiments (*p* < 0.0001). No significant differences were observed for conical IACs in the random versus flat IACs in the best‐case experiments (*p* = 1.000) and for conical IACs in the best‐case versus flat IACs in the random experiments (*p* = 0.982).

**Table 5:** Other intergroup differences.

Comparison across connection types and assembly scenarios					*p* value
Conical	Random	vs.	Flat	Best case	1.000
Conical	Worst case	vs.	Flat	Best case	0.0001*
Conical	Random	vs.	Flat	Worst case	0.0001*
Conical	Best case	vs.	Flat	Worst case	0.0001*
Conical	Best case	vs.	Flat	Random	0.982
Conical	Worst case	vs.	Flat	Random	0.0001*

* Differences that are statistically significant.

## 4 Discussion

The results of this in vitro study show that the rotational stability of rotationally nonsymmetrical abutments did affect vertical‐dimension changes of implant‐supported restorations. Hence the first null hypothesis was rejected, given significant differences in vertical‐dimension values. The second null hypothesis could be sustained, considering that the vertical‐dimension changes did not significantly differ depending on which IAC design (conical or flat) was used.

In the process of analyzing vertical‐dimension changes of the implant‐supported three‐unit FPDs, we observed significant differences in rotational stability. Significantly less favorable values were measured for the worst‐case than for the best‐case or the random assembly scenario. Several in vitro studies have evaluated the rotational freedom of IACs against the resultant prosthetic restorations. Movements within the IAC of single‐tooth restorations have been reported to potentially result in screw loosening and superstructure damage (Binon 1995; Binon and McHugh 1996). Some authors have reported that, to ensure optimal stability of the screw connection, the fit between the external hexagon of the implant and the internal hexagon of the abutment should have less than 5° of rotational freedom (Semper, Heberer, et al. 2010; Binon and McHugh 1996; Lang et al. 2003).

Past studies have generally used experimental setups that were largely confined to investigating the effects of repeated manual disassembly and reassembly on position stability (Semper, Heberer, et al. 2010; Semper‐Hogg et al. 2013). Since the present in vitro study is, to our knowledge, the first of its kind to simulate a clinical process in its entirety and to investigate differential effects of aligning implant components with either the left or the right stop of the positional index, its results cannot be readily compared to any of the studies documented in the literature.

In our best‐case experiments, all components were assembled to be aligned with the right‐limit stop of the positional index, whereas the worst‐case experiments, characterized by alternating index positions, gave rise to substantial deviations on the stone casts compared to the true situations on the aluminum models. These FPDs tended to have an exaggerated vertical dimension, which, in daily clinical practice, would call for extensive occlusal adjustments by the clinician or even for a remake. Future studies should look into the consequences of assembling impression posts, laboratory analogs, and abutments to different index positions.

Lastly, our third experimental scenario of random individuals characterized by different levels of expertise in implant dentistry and prosthodontics manually assembling the components included five experienced (> 5 years) and five less experienced (< 5 years) dentists. This random group was found to produce values somewhat, but not significantly, higher than the best‐case group. The mean vertical dimensions measured in both groups were of a magnitude requiring little or no occlusal adjustments by the dentist. Nevertheless, higher maximum values were observed in the random than in the best‐case group, presumably due to the less experienced dentists or to lower degrees of rotational stability of the IACs. The minimum values in the random group, by contrast, were similar to the minimum values in the best‐case group.

All of the implant components used in this study are associated with manufacturing tolerances that will affect rotational stability no matter what type of positional index is selected (Semper‐Hogg et al. 2013). Accurate details on these tolerances are not provided by the manufacturers.

On comparing our results for these ICE designs to previous studies, it emerges that higher vertical‐dimension changes have, in the past, been reported with conical than with flat IACs (Semper, Heberer, et al. 2010; Semper‐Hogg et al. 2013). Conical connections will naturally involve a vertical play essential to their frictional fit. According to one study, they do not reliably preserve the vertical dimension of implant‐supported superstructures and can result in considerable imprecisions of fit (Semper, Heberer, et al. 2010). In addition, differences across manufacturers also exist within conical connections (Semper‐Hogg et al. 2013). Aside from manufacturing tolerances, torque values can make a difference as well (Dailey et al. 2009), and manual tightening of the abutment screw to the point of definitive insertion carries a risk of deviations caused by highly variable torque values (Kanawati et al. 2009).

In our best‐case group of assembly experiments, the implant system with a conical IAC yielded values only slightly lower than the flat‐IAC implant system. Hence, the possibility of conical connections being more susceptible to vertical‐dimension changes did not leave a significant mark on our findings. Nor did the different IAC designs involve major differences in minimum and maximum values. Therefore, unlike previous authors, we do not believe that any vertical‐dimension changes contributed by conical IACs would be significant enough to affect the fit of the definitive prosthetic restoration.

The present in vitro study has several strengths. Its simulation of the entire clinical and laboratory process, from impression‐taking to intraoral delivery, may serve as a blueprint for future studies. As for limitations, only one conical and one flat IAC from a single manufacturer were included. Due to different manufacturing tolerances, further studies are needed, including the use of digital impression‐taking and modeling.

Our results are of everyday interest to clinicians involved in implant dentistry, and to prosthodontists especially, given the challenges associated with the vertical dimension of any restoration. Even minute deviations in this regard will constitute a major problem for patients, and while clinicians will routinely perform occlusal adjustments after intraoral delivery, there are limits to what this can achieve. To counteract the risk of having to remake entire restorations for this reason, it is useful to consider the main finding of the present study, that is, that the vertical dimension of a multi‐unit restoration will be affected not so much by different IAC designs as by different rotational positions of the various implant components attached throughout the clinical and laboratory fabrication process.

Thus our recommendation is to effectively minimize these rotational differences with a view to keeping the vertical dimension of a restoration as stable as possible. Of course, an IAC design with low rotational freedom will, by itself, reduce any vertical discrepancies sustained over the course of repeatedly assembling, disassembling, and reassembling a number of different implant components (impression posts, laboratory analogs, abutments). The present study, however, adds to this inherent quality by demonstrating that a strict protocol of attaching any of these components in alignment with the right‐limit stop of the positioning index can stabilize the vertical dimension even further.

## 5 Conclusion

From the results of this in vitro study, the conclusion can be drawn that the vertical height of restorations is not significantly influenced by whether a conical or a flat IAC is selected. Rotational stability made a greater and statistically significant difference to the vertical integration of an implant‐supported FPD than the IAC design. Judging from this in vitro study, it is recommended to pursue an assembly strategy whereby impression posts, laboratory analogs, and abutments are invariably attached in alignment with the right‐limit stop of the positioning index. A basal, and not an occlusal, view should be taken on the positioning of laboratory analogs for impression‐taking.

## Author Contributions

Holger Zipprich and Silvia Brandt designed the study, analyzed the data, and drafted the original manuscript. Stefanie Ecker conducted the experimental setup. Holger Zipprich and Stefanie Eckert performed the statistical analysis. Pauline Gutmann and Kathrin Seidel reviewed the statistical analysis and performed the visualization of the experimental setup. Paul Weigl and Markus Schlee analyzed the data and critically revised the manuscript. All authors approved the final version to be published.

## Conflicts of Interest

The authors declare no conflicts of interest.

## Data Availability

The data that support the findings of this study are available on request from the corresponding author. The data are not publicly available due to privacy or ethical restrictions.
